# Dexmedetomidine Inhibits Maturation and Function of Human Cord Blood-Derived Dendritic Cells by Interfering with Synthesis and Secretion of IL-12 and IL-23

**DOI:** 10.1371/journal.pone.0153288

**Published:** 2016-04-07

**Authors:** Gong Chen, Yuan Le, Lei Zhou, Li Gong, Xiaoxiao Li, Yunli Li, Qin Liao, Kaiming Duan, Jianbin Tong, Wen Ouyang

**Affiliations:** 1 Department of Anesthesiology, the Third Xiangya Hospital of Central South University, Changsha, Hunan, China; 2 Center for Experimental Medicine, the Third Xiangya Hospital of Central South University, Changsha, Hunan, China; University of Pennsylvania, UNITED STATES

## Abstract

**Aims:**

To investigate the effects and underlying mechanism of dexmedetomidine on the cultured human dendritic cells (DCs).

**Methods:**

Human DCs and cytotoxic T lymphocytes (CTLs) were obtained from human cord blood mononuclear cells by density gradient centrifugation. Cultured DCs were divided into three groups: dexmedetomidine group, dexmedetomidine plus yohimbine (dexmedetomidine inhibitor) group and control group. DCs in the three groups were treated with dexmedetomidine, dexmedetomidine plus yohimbine and culture medium, respectively. After washing, the DCs were co-incubated with cultured CTLs. The maturation degree of DCs was evaluated by detecting (1) the ratios of HLA-DR-, CD86-, and CD80-positive cells (flow cytometry), and (2) expression of IL-12 and IL-23 (PCR and Elisa). The function of DCs was evaluated by detecting the proliferation (MTS assay) and cytotoxicity activity (the Elisa of IFN-γ) of CTLs. In addition, in order to explore the mechanisms of dexmedetomidine modulating DCs, α2-adrenergic receptor and its downstream signals in DCs were also detected.

**Results:**

The ratios of HLA-DR-, CD86-, and CD80-positive cells to total cells were similar among the three groups (*P*>0.05). Compared to the control group, the protein levels of IL-12 and IL-23 in the culture medium and the mRNA levels of IL-12 p35, IL-12 p40 and IL-23 p19 in the DCs all decreased in dexmedetomidine group (*P*<0.05). In addition, the proliferation of CTLs and the secretion of IFN-γ also decreased in the dexmedetomidine group, compared with the control group (*P*<0.05). Moreover, these changes induced by dexmedetomidine in the dexmedetomidine group were reversed by α2-adrenergic receptor inhibitor yohimbine in the dexmedetomidine plus yohimbine group. It was also found the decrease of mRNA levels of IL-12 p35, IL-12 p40 and IL-23 p19 in the dexmedetomidine group could be reversed by ERK1/2 or AKT inhibitors.

**Conclusion:**

Dexmedetomidine could negatively modulate human immunity by inhibiting the maturation of DCs and then decreasing the proliferation and cytotoxicity activity of CTLs. The α2-adrenergic receptors and its downstream molecules ERK1/2 and AKT are closely involved in the modulation of dexmedetomidine on DCs.

## Introduction

Tumors are one of the most important health problems of human. Much work has been done to investigate the methods of prevention and treatments of tumors. So far, surgical excision remains the main treatment for solid tumors [1, 2]. However, recent studies have shown that perioperative treatments obviously affect the immunity and the recurrence and metastasis of tumors after surgery [3]. For example, anesthetic isoflurane enhanced growth and malignant potential of cultured prostate, renal and ovarian cancer cells via activating hypoxia-inducible factor (HIF) signaling pathway [4–7]; Surgical stress could inhibit immunity via modulating natural killer (NK) cells and T lymphocytes [3, 8, 9], and finally increased the survival of circulated tumors cells [10, 11]. Thus much attention is paid to the immunity modulation of perioperative treatments of tumor patients.

Dendritic cells (DCs) are antigen presenting cells and play a pivotal role in perioperative anti-tumor immunity [12]. The anti-tumor function of DCs is mainly depended on the maturation status. Immature DCs facilitate tumor tolerance while mature DCs promote anti-tumor immunity. For example, in breast cancer patients mature DCs of metastatic lymph nodes were less than that of non-metastatic lymph nodes [13]. The mature DCs can secret immunostimulating cytokines IL-12 and IL-23 [14]. IL-12 enhances cytotoxic effect of NK cells and CD8^+^ T lymphocytes [15]. IL-23 promotes Th17 cells to recruit other immune cells into tumors [16, 17]. In addition, mature DCs can activate cytotoxic T lymphocytes (CTLs) to directly kill tumor cells [18]. Thus, perioperative drugs that modulate the maturation of DCs may influence anti-tumor immunity and metastatic recurrence.

Dexmedetomidine (DEX) is often used as a clinical sedative commonly used in anesthesia and intensive care unit. It functions via α2-adrenoceptors of presynaptic membrane of nerve cells. It has less inhibition on respiration and less risk to induce delirium relative to other sedatives [19]. Thus, it is becoming more and more popular in the tumorectomy anesthesia and postoperative pain analgesia in tumor patients. However, recently, it is reported that intraperitoneal injection with DEX increased growth and metastasis of implanted breast tumor in mice [20]. In addition, Reeteka Sud detected the expression of α2-adrenoceptors on immune cells such as monocytes and macrophages [21]. DEX was reported to suppress the phagosome proteolysis and migration of murine bone marrow-derived DCs through α2-adrenoceptors [22]. These research suggest that DEX may influence perioperative immunity of tumor patients, and affect their prognosis. However, the effect of anesthetics on DCs is variable from different species. For example, opioids suppress maturation and function of murine DCs but enhance maturation and function of human DCs [23–25]. Therefore here we investigated the influences of DEX on human cord blood-derived DCs via testing maturation of DCs and detecting proliferation and cytotoxicity of CTLs co-cultured with DCs.

## Materials and Methods

### Reagents and apparatus

X-VIVO™ 15 serum-free specialty cell culture medium (catalog number: 04-418Q) was purchased from Lonza (Switzerland). DMSO (catalog number: d5879), yohimbine (catalog number: 731242), PD98059 (catalog number: P215), LY294002 (catalog number: L9908), SB203580 (catalog number: S8307) and FITC conjugated dextran (catalog number: FD40S) were purchased from Sigma (USA). Ficoll Pague PLUS (catalog number: 17-1440-03) was obtained from GE (USA), rh GM-CSF (recombinant human GM-CSF, catalog number: AF-300-03), rh IL-4 (catalog number: AF-200-04), rh TNF-α (catalog number: AF-300-01A) and rh IL-2 (catalog number: 200–02) were provided by Peprotech (Britain). PE or APC conjugated mouse-anti-human monoclonal antibody used for the detection of CD 11c, HLA-DR, CD86, CD80 were purchased by Biolegend (USA). Human IL-12 (catalog number: CSB-E04599h), IL-23 (catalog number: CSB-E08461h) and IFN-γ (catalog number: CSB-E04577h) Elisa kit were obtained by cusabio (China). The α2A rabbit-anti-human antibody (catalog number: ab65833) was purchased by Abcam (Britain). Reagent of dexmedetomidine was provided by Hengrui Medicine Company (China).

### Induction of cord blood dendritic cells and cytotoxic T lymphocytes

The research was approved by the Institutional Review Board of the Third Xiangya Hospital of Central South University and was registered with the Chinese Clinical Trial Registry (ChiCTR-IPR-14005271). Cord blood (60-80ml) was obtained from each healthy puerperant informed by written consent from May 2014 to April 2015. Cord blood diluted with normal saline (1:1) was added on the surface of the Ficoll and then centrifugated with 400G for 25 minutes at 20°C. Mononuclear cells of cord blood were taken out from the interlayer and cultured in the serum-free medium for 2hrs at 37°C. Cultured cells were then separated into adherent cells and suspension cells.

In order to get the DCs, adherent cells were further cultured in X-VIVO™ 15 serum-free medium plus rh GM-CSF (100ng/ml) and rh IL-4 (100ng/ml). Fresh serum-free medium plus rh GM-CSF and IL-4 (both 100ng/ml) were added into the medium on the 3^rd^ and 5^th^ day, respectively. Immature DCs were harvested on the 6^th^ day. On the 7^th^ day, rh TNF-α (100ng/ml) was also added to induce maturation of DCs. On the 8^th^ day, mature DCs were collected for further analysis and co-culture with CTLs.

In order to get the CTLs, the suspension cells were transferred to another culture flask and further cultured in X-VIVO™ 15 serum-free medium plus 1000U/ml rh IL-2. Fresh serum-free medium plus 1000U/ml IL-2 were replenished on the 3^rd^, the 5^th^ and the 7^th^ days. On the 8^th^ day, CTLs were matured and collected.

On the 8^th^ day, mature DCs and CTLs were mixed together and were cultured 3 more days for mixed lymphocyte reaction (MLR). The purpose of MLR assay was for the test of proliferation activity and IFN-γ level of co-cultured CTLs.

### Detection of DC phenotypes

DCs were labeled by PE-conjugated CD11c and CD86, or APC-conjugated HLA-DR and CD80 for 40 minutes at 4°C, according to the manual instruction. After three washes in PBS, DCs were fixed in 4% paraformaldehyde PBS solution, then washed 3 times and finally detected by flow cytometry FC500 (Bechman coulter). Immature DCs were characterized by high expression of CD11c and HLA-DR, but relative low expression of CD86 and CD80. While mature DCs were characterized by high expression of CD86 and CD80. In order to detect the expression of α2-adrenergic receptors on the cell surface, DCs were incubated in solution of rabbit-anti-human antibody of α2A receptor for 2hrs. After three washes, DCs were further incubated in solution of CY3-conjugated goat-anti-rabbit antibody. The positive staining of α2A receptor was detected by immunofluorescence.

### Western blotting

Western blotting was used to assess the expression of ERK1/2/p-ERK1/2, p38/p-p38, AKT/p-AKT and β-actin in the cultured DCs. The protocol was as the following. Briefly, DCs were collected and homogenized in a lysis buffer containing protease inhibitors cocktails (Roche, Germany, catalog number: P8340) and phenylmethanesulfonylfluoride (PMSF, Sigma, USA, catalog number: p7626). The quantity of protein of samples was determined using a BCA protein assay kit (Wellbio, China) according to the manufacturer’s instructions. Equal amounts of protein samples (/lane) were separated by sodium dodecyl sulfate polyacrylamide gel electrophoresis (SDS-PAGE) and transferred to polyvinylidene fluoride membranes. Membranes were blocked with 5% skim milk in TBST buffer for 1 hr and then incubated with primary antibodies (rabbit monoclonal antibody to ERK1/2: 1:1000, CST, USA, catalog number #4695S; rabbit monoclonal antibody to p-ERK1/2: 1:1000, CST, USA, catalog number #4370S; rabbit polyclonal antibody to AKT: 1:500, Proteintech, USA, catalog number 10176-2-AP; rabbit monoclonal antibody to p-AKT: 1:1000, CST, USA, catalog number #4060P; rabbit monoclonal antibody to p38: 1:1000, CST, USA, catalog number #8690S; rabbit polyclonal antibody to p-p38: 1:1000, Bioworld, USA, catalog number BS4766; rabbit monoclonal antibody to β-actin: 1:4000, proteintech, USA, catalog number 60008-1-ig) overnight at 4°C. After three washes, membranes were incubated with the secondary antibodies (1:3000) at room temperature for 60min. Finally, visualization of the proteins was accomplished by enhanced chemiluminescence detection kit (Pierce; Thermo Scientific, Shanghai, China), and the intensity of each band was quantified by densitometry. Relative expression levels of protein were normalized by the ratio of target protein to β-actin.

### Real-time quantitative PCR

Total RNA was isolated from the DCs by Trizol reagent (Invitrogen, Barcelona, Spain), according to the manufacturer’s instructions. The RNA concentration and quality were determined with NanoDrop spectrophotometer Nano-200 (Thermo Scientific, Wilmington, DE). Real-time PCR reactions were run in triplicate for each sample on a Bio-Rad MyCycler iQ5. Primer sequences were designed using Beacon Designer software (version 7.2, PREMIER Biosoft International, Palo Alto, CA) and thoroughly tested. The primers used in the experiment were listed as follows: human IL-12 p35 (Forward primer: GCT CCA GAA GGC CAGA CAAA; Reverse primer: GCC AGG CAA CTC CCAT TAGT); human IL-12 p40 (Forward primer: ACC TGA CCC ACC CAAG AACTT; Reverse primer: TGG ACC TGA ACGC AGAA); human IL-23 p19 (Forward primer: CTC TGC TCC CTG ATAG CCCT; Reverse primer: TGC GAA GGA TTT TGAA GCGG); human alpha 2A (α2A receptor) (Forward primer: GGT GTT ATG AAG TCC CTC TATG; Reverse primer: GAA AAG GCA ATT ATG CTG TTAG); human alpha 2B (α2B receptor) (Forward primer: GTA GAC TTT TGT TCT GTC CCTG; Reverse primer: TAG CGT AAT AAC TCA GAC CTTT); human alpha 2C (α2C receptor) (Forward primer: GCT GTG AGG TCA GGG TTT TAG; Reverse primer: GAT TGT CGG TGC TTT CTC CTT); β-actin (Forward primer: CAT CCT GCG TCT GGAC CTGG; Reverse primer: TAA TGT CAC GCA CGAT TTCC). In brief, 1 μg of total RNA was reverse-transcribed using the cDNA Synthesis Kit (Fermentas). The PCR reaction was run for 30 cycles for 94°C(30s), 60°C(30s), 72°C(30s). After amplification, a threshold was set for each gene and Ct values were calculated for all samples. Gene expression changes were analyzed using the built-in iQ5 Optical system software. The results were normalized using a reference gene, β-actin, determined with Genex software as the most stable for the treatment conditions used. For the RT-PCR, the products were separated in 1% agarose gels. DNA bands were visualized with ethidium bromide and analyzed using UNSCANIT software package.

### ELISA

Concentrations of IL-12, IL-23 and IFN-γ in the supernatant of DCs and CTLs were measured using ELISA kits (cusabio, China) according to the manufacturer’s protocol. Developed color reaction was measured as OD units at 450 nm. The detectable concentration ranges were 4.7 pg/ml-300 pg/ml for human IL-12; 4.7 pg/ml-300 pg/ml for human IL-23; 6.25 pg/ml-400 pg/ml for human IFN-γ.

### Endocytosis assay with FITC-dextran

The endocytosis assay was performed as previously described [26, 27]. Immature DCs (1×10^6^ cells/ml) collected on the 6^th^ day were incubated in X-VIVO™ 15 serum-free medium plus FITC-dextran (final concentration: 50μg/ml) and DEX (1ng/ml, 2ng/ml and 4ng/ml respectively) at 37°C for 30 minutes. Endocytosis of the tracer was stopped at the indicated time points by rapidly cooling the cells on ice. After washing three times with cold PBS, the cells were detected via flow cytometry FC500 (Bechman coulter). Incubation of cells with FITC-dextran on ice was used as a background control. The mean fluorescence intensity (MFI) resulting from the subtraction of background control from each experimental sample represented the quantity of incorparated tracer. To examine the effects of α2-adrenoceptor inhibitors on endocytosis, cells were pretreated with yohimbine (4ng/ml, 8ng/ml and 16ng/ml respectively) at 37°C for 30 min. After yohimbine pretreatment, the cells were incubated with the endocytic tracer and DEX in the presence of yohimbine.

### Mixed lymphocyte reaction

DCs and CTLs (DCs/CTLs is 1:5) were mixed and then co-cultured in flasks for 3days. In order to analyze cell metabolic rate via MTS (3-(4,5-dimethylthiazol-2-yl) -5- (3-carboxy methoxyphenyl) -2- (4-sulfophenyl)-2H-tetrazolium) assay, 100μl cell suspension of mixed cells from every flask was transferred into 96-well plates, and then 10μl MTS solution was added into each well of 96-well plates. Finally, the 96-well plates were kept at 37°C for 1-4hrs and were detected the OD at 490nm using spectrometer.

### Statistical analysis

Data were showed as mean±SD. Data were analyzed using the Student’s t-test. P<0.05 was considered statistically significant. All statistical analysis was performed using SPSS 19.0 software.

## Results

### 1. Dexmedetomidine inhibits maturation and function of human cord blood-derived DCs

**Fig 1:**
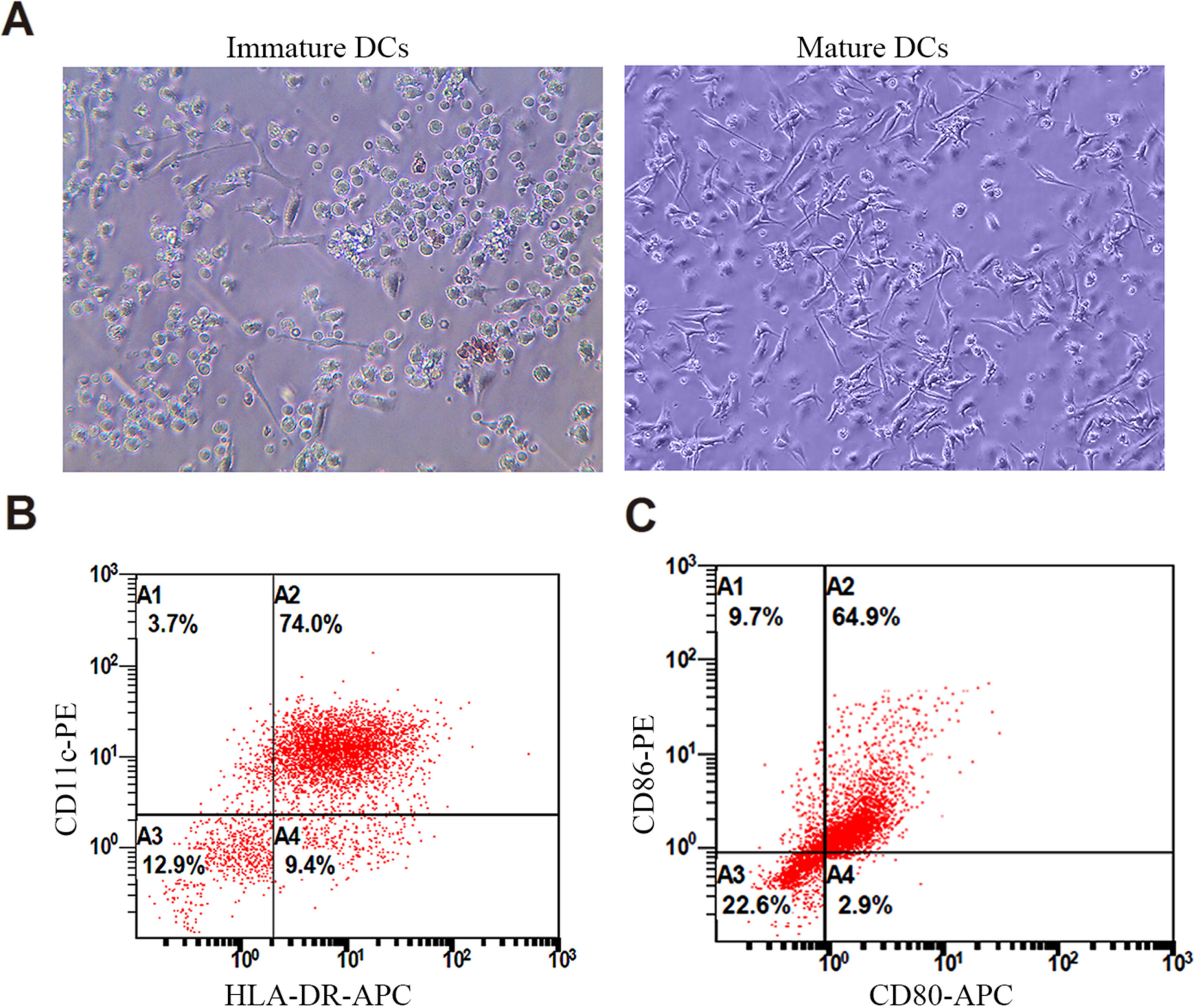
Identification of human DCs. (A) Examples of immature and mature DCs. Immature DCs showed multiple spiculated edges. Mature DCs were veiled or dendritic spine-like at semi-suspension state; (B) Flow cytometry identification of mature DCs: the ratios of CD11c-, HLA-DR-, CD86-, and CD80-positive cells to total cells were 77.7%, 83.4%, 74.9% and 67.8%, respectively.

Mature DCs were usually characterized by the high expression of DC-specific marker CD11c, major histocompatibility complex molecule HLA-DR, and costimulatory molecules CD80 and CD86 [23, 27]. In addition, mature DCs were at semi-suspension state with the shape of veiled or dendritic spine morphology. In contrast, immature DCs showed the characteristic multiple spiculated edges [18]. Here we followed the above standards to identify human cord blood-derived DCs. Similar to previous reports [18], immature human DCs appeared with multiple spiculated edges (Fig 1A). Matured DCs were veiled or dendritic spine-like at semi-suspension state (Fig 1A). 77.7% of mature DCs expressed high amounts of CD11c, 83.4% expressed HLA-DR, 74.9% expressed CD86, and 67.8% expressed CD80 (Fig 1B). The positive ratios were similar with previous reports [18, 28].

**Fig 2:**
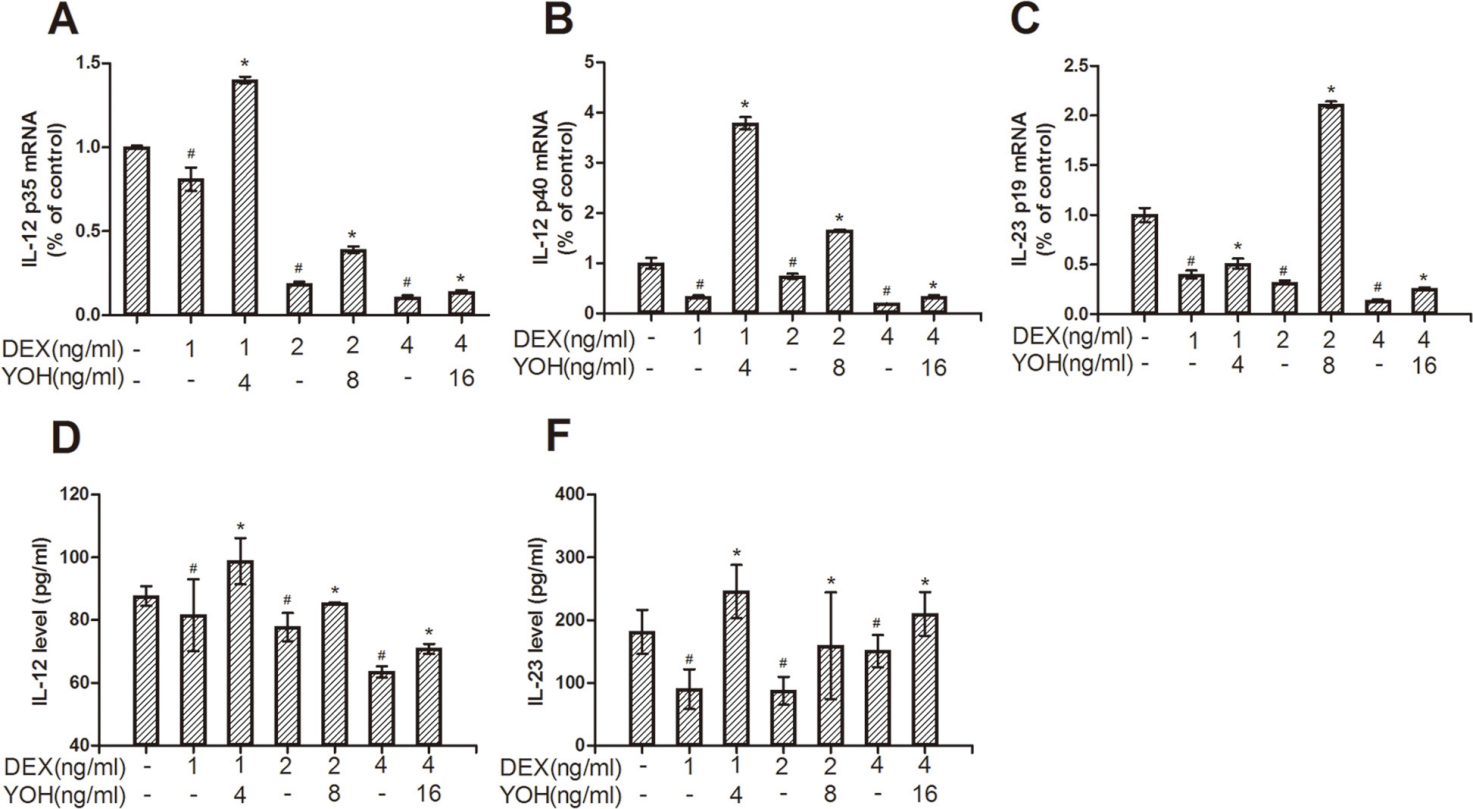
Effect of DEX on IL-12 and IL-23 production in mature DCs. (A), (B) and (C) showed mRNA levels of IL-12 p35, IL-12 p40 and IL-23 p19, respectively. (D) and (E) showed the protein levels of IL-12 and IL-23, respectively. “DEX” is short for dexmedetomidine, “YOH” is short for yohimbine. Immature DCs were differentiated in the absence or presence of different doses of DEX (1ng/ml, 2ng/ml and 4ng/ml) and yohimbine (4ng/ml, 8ng/ml and 16ng/ml) for 1 day before matured with TNF-α (“-”means do not add DEX or yohimbine). Grouping is consistent in these bar charts. The data were expressed as mean±SD of the results from these experiments. #: *P*<0.05, compared to the control group; *: *P*<0.05, compared to the relative DEX only group.

Based on the cultured DCs, we further investigated the effect of DEX on the maturation and function of DCs. The maturation of DCs was analyzed via examining (1) the ratios of HLA-DR-, CD86-, and CD80-positive cells and (2) the secretion of IL-12 and IL-23. We found that the ratios of HLA-DR-, CD86-, and CD80-positive cells to total cells were similar among the control group, DEX group and DEX plus yohimbine group (*P*>0.05) (data not shown). However, compared to the control group, the protein levels of IL-12 and IL-23 in the culture medium of DCs decreased dose-dependently in DEX group (*P*<0.05) (Fig 2D and 2E, S1 Table). To further investigate the effect of DEX on these two proinflammatory cytokines, we analyzed the mRNAs of IL-12 p35, IL-12 p40 and IL-23 p19 by real-time PCR. IL-12 is composed of subunits IL-12 p35 and IL-12 p40. And IL-23 is composed of subunits IL-23 p19 and IL-12 p40. Thus IL-12 p40 is the common subunit of IL-12 and IL-23. Compared to the control group, the mRNA levels of IL-12 p35, IL-12 p40 and IL-23 p19 were decreased in DEX group (*P*<0.05) (Fig 2A, 2B and 2C, S1 Table). These showed that DEX inhibited the maturation of DCs.

**Fig 3:**
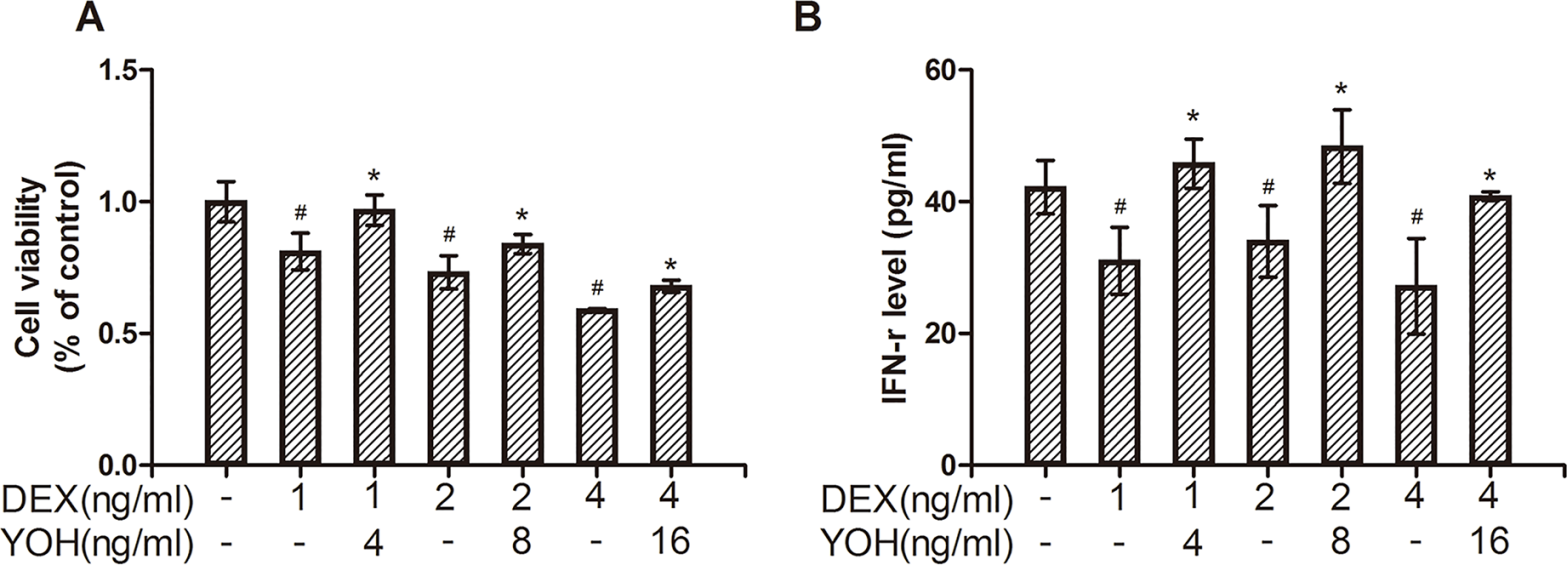
Effects of DEX-treated DCs on co-cultured CTLs proliferation and IFN-γ production. (A) Showing the CTLs proliferation via MTS assay. (B) Showing IFN-γ secretion of CTLs via Elisa detection. CTLs were co-cultured with DCs pretreated with different doses of DEX or yohimbine. The data were expressed as mean±SD. #: *P*<0.05, compared to the control group; *: *P*<0.05, compared to the relative DEX only group.

The function of DCs was analyzed via endocytosis assay of immature DCs, the proliferation assay and IFN-γ secretion of co-cultured CTLs. We found that the MFI (mean fluorescence intensity) of endocytic FITC-dextran detected by flow cytometry was similar among the control group, DEX group and DEX plus yohimbine group (*P*>0.05) (data not shown). Thus, DEX did not show notable effect on endocytosis function of immature DCs. However, compared with the control group, the proliferative activity of co-cultured CTLs decreased dose-dependently in DEX group (*P*<0.05) (Fig 3A, S2 Table). Meanwhile, compared to the control group, the protein level of IFN-γ in the medium of co-cultured CTLs also decreased in DEX group (*P*<0.05) (Fig 3B, S2 Table). These showed that DEX also inhibited the function of mature DCs.

### 2. α2-adrenoceptors and downstream ERK1/2 and AKT signals were closely involved in the modulation of DEX on DCs

**Fig 4:**
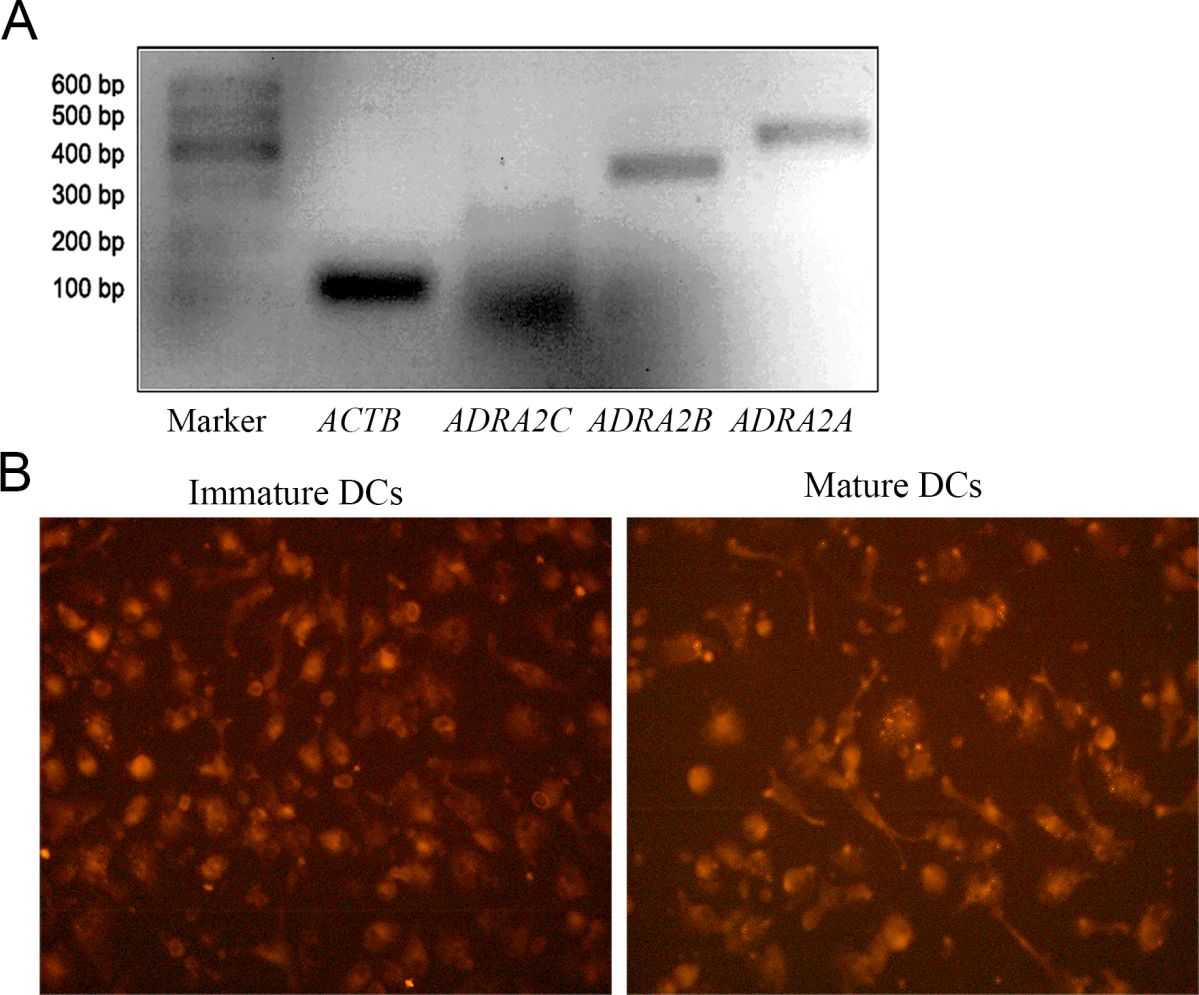
Expression of α2-adrenoceptors. (A) mRNA expressions of α2A and α2B receptors in DCs. Abbreviations: *ADRA2A* (α2A receptor); *ADRA2B* (α2B receptor); *ADRA2C* (α2C receptor); *ACTB* (actin beta); (B) immunostaining of α2A receptors of immature DCs and mature DCs (magnification ×400).

DEX is an agonist of α2-adrenoceptor. In order to explore the possible mechanisms of DEX’s modulation on perioperative immunity, we first examined the expression of α2-adrenoceptors on human DCs by RT-PCR and immunofluorescence. In line with the study of murine DCs [22, 26], expression of α2-adrenoceptors was also detected in human DCs (Fig 4).

Yohimbine is an antagonist of α2-adrenoceptor. In order to determine the mechanism of DEX, we compared the difference of maturation and function of DCs among the normal control, DEX treatment and DEX plus yohimbine treatment groups. We found that compared to the normal control, DEX treatment decreased (1) protein levels of IL-12 and IL-23 and (2) mRNA levels of IL-12 p35, IL-12 p40 and IL-23 p19 of DCs (Fig 2, S1 Table). These indicated that DEX inhibited maturation of human DCs. However, compared to the DEX group, α2-adrenoceptor antagonist yohimbine reversed DEX-induced decreases of (1) protein levels of IL-12 and IL-23 (*P*<0.05) (Fig 2D and 2E, S1 Table) and (2) mRNA levels of IL-12 p35, IL-12 p40 and IL-23 p19 (*P*<0.05) (Fig 2A, 2B and 2C, S1 Table) in DEX plus yohimbine group. These showed that DEX inhibited maturation of human DCs via α2-adrenoceptors. In addition, DEX-treated DCs could inhibit proliferation of CTLs (Fig 3A, S2 Table) and IFN-γ level of CTLs co-cultured with DCs (Fig 3B, S2 Table), although normal DCs didn’t have similar function. These indicated that DEX also inhibited function of human DCs. Moreover, α2-adrenoceptor inhibitor yohimbine could reverse DEX-induced changes of cell proliferation (Fig 3A, S2 Table) and IFN-γ level (Fig 3B, S2 Table) of CTLs. Thus, DEX inhibited the maturation and function of DCs possibly through α2-adrenoceptors.

**Fig 5:**
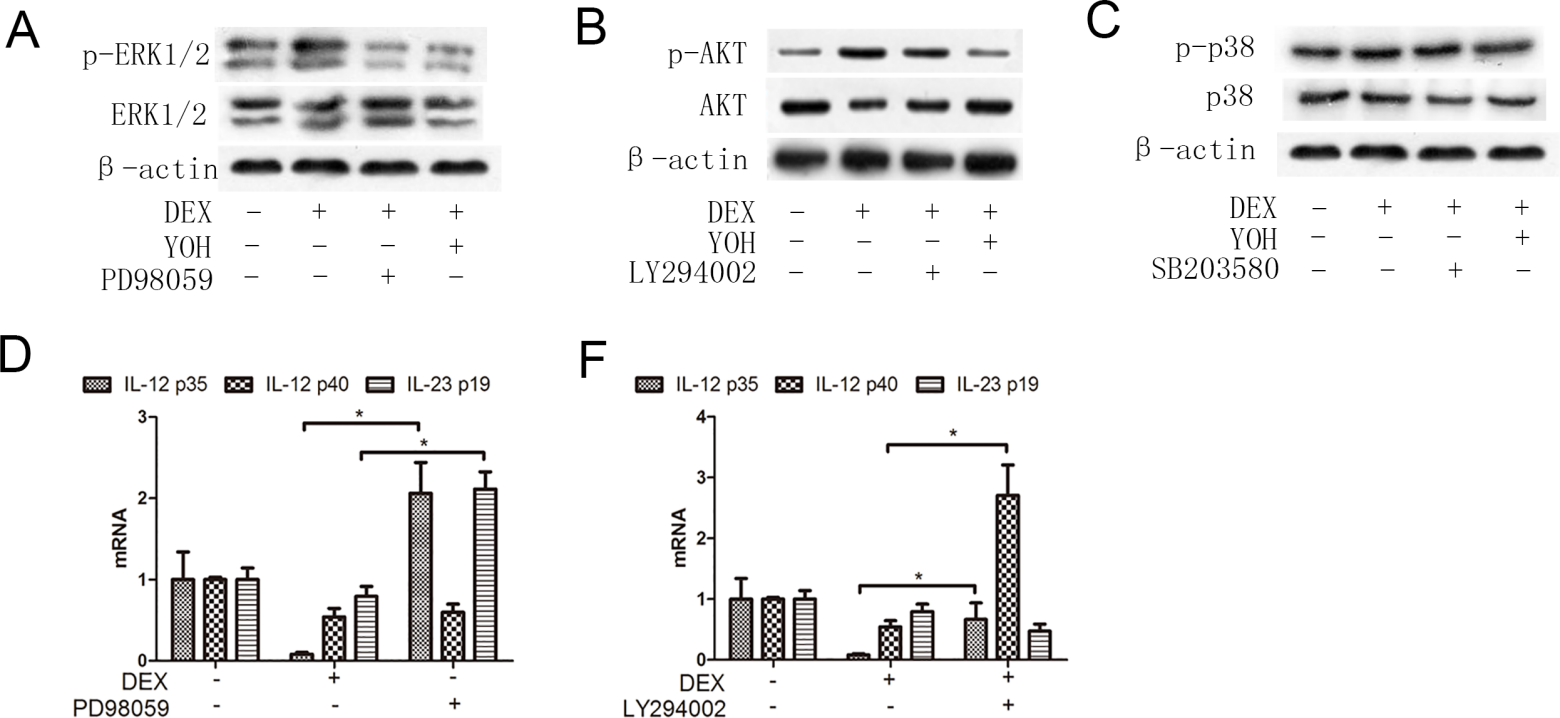
ERK1/2, AKT and p38 in human DCs. (A) ERK1/2 and p-ERK1/2 expressions: immature DCs were cultured in the absence or presence of DEX (2ng/ml), yohimbine (8ng/ml) and PD98059 (40μM). After 30 min, cells were harvested to test protein levels of ERK1/2 and p-ERK1/2; (B) AKT and p-AKT expressions: immature DCs were cultured in the absence or presence of DEX (2ng/ml), yohimbine (8ng/ml) and LY294002 (30μM). After 30 min, cells were harvested to test protein levels of AKT and p-AKT; (C) p38 and p-p38 expressions: immature DCs were cultured in the absence or presence of DEX (2ng/ml), yohimbine (8ng/ml) and SB203580 (30μM). After 30 min, cells were harvested to test protein levels of p38 and p-p38. (D) Immature DCs were cultured in the absence or presence of PD98059 (40Μ) for 30 min, followed by treatment with DEX (2ng/ml) for 30 min, then matured with TNF-α (100ng/ml). After 24hrs, cells were collected to test mRNA levels of IL-12 p35, IL-12 p40 and IL-23 p19; (E) Immature DCs were cultured in the absence or presence of LY294002 (30Μ) for 30 min, followed by treatment with DEX (2ng/ml) for 30 min, then matured with TNF-α (100ng/ml). After 24hrs, cells were collected to test mRNA levels of IL-12 p35, IL-12 p40 and IL-23 p19. The results are presented as mean±SD. *P<0.05.

AKT, ERK1/2 and p38 are main downstream signal molecules of α2-adrenoceptors activation in mouse [26, 29–31]. In order to determine whether these molecules were the targets of DEX in human DCs, we compared total protein levels and activation of AKT, ERK1/2 and p38 (active forms of these molecules were p-AKT, p-ERK1/2 and p-p38, respectively) among the normal control, DEX treatment and DEX plus yohimbine treatment groups. We found that levels of total AKT, ERK1/2 and p38 molecules were similar among these three groups. However, compared to the normal control, DEX treatment significantly enhanced p-AKT and p-ERK1/2 levels of DCs (Fig 5A and 5B). And blocking α2-adrenoceptors by inhibitor yohimbine, however, reversed DEX-induced enhancement of p-AKT and p-ERK1/2 levels in DEX plus yohimbine treatment group (Fig 5A and 5B). In contrast, DEX and yohimbine showed little or no effect on p-p38 levels (Fig 5C). These suggested that p-ERK1/2 and p-AKT were the possible targets of DEX in human DCs.

The up-regulation of IL-12 and IL-23 production is one of the characteristic of DC maturation. To further confirm the roles of ERK1/2 and AKT signals on DEX’s modulation in human DCs, we examined mRNA levels of the subunits IL-12 p35, IL-12 p40 and IL-23 p19 among the normal control, DEX treatment and DEX plus signal inhibitor groups (PD98059 is the inhibitor of p-ERK1/2; LY294002 is the inhibitor of p-AKT). We found that compared to the normal control, DEX treatment decreased mRNA levels of IL-12 p35, IL-12 p40 and IL-23 p19 of DCs (*P*<0.05) (Fig 5D and 5E, S3 Table). And blocking ERK1/2 signal with inhibitor PD98059 significantly reversed DEX-induced decreases of IL-12 p35 and IL-23 p19 in DEX plus PD98059 treatment group (*P*<0.05) (Fig 5A and 5D, S3 Table). Similar to the role of ERK1/2, p-AKT inhibitor LY294002 also reversed DEX treatment-induced decreases of IL-12 p35 and IL-12 p40 (*P*<0.05) (Fig 5B and 5E, S3 Table). Thus p-ERK1/2 and p-AKT were the main downstream signals of DEX’s modulation in human DCs.

## Discussion

The purpose of our study was to investigate the effects and mechanisms of dexmedetomidine (DEX) on the cultured human dendritic cells (DCs). We demonstrated that DEX suppressed the production and secretion of IL-12 and IL-23 from human DCs, and decreased cell proliferation rate and IFN-γ secretion in CTLs co-cultured with DCs. We also found that human DCs expressed α2-adrenoreceptors, and inhibiting α2-adrenoreceptor by inhibitor yohimbine reversed the effects of DEX on DCs and co-cultured CTLs. Further study showed that α2-adrenoreceptor inhibitor yohimbine could inhibit DEX-induced activation of α2-adrenoreceptor downstream signal molecules ERK1/2 and AKT in DEX-treated DCs. ERK1/2 inhibitor could reverse the inhibition of DEX on expression of IL-12 p35 and IL-23 p19, and AKT inhibitor could reversed the inhibition of DEX on expression of IL-12 p35 and IL-12 p40. These results suggest that DEX inhibits the maturation and function of DCs via α2-adrenoreceptors and its downstream signals ERK1/2 and AKT.

Perioperative anesthesia significantly affects the recurrence and metastasis of tumors after surgery [32–35]. Previous studies showed that compared to the general anesthesia, paravertebral anesthesia obviously reduced the risk of recurrence and metastasis of breast cancer during the initial 36 months after surgery [36]. Further studies showed that anesthesia could obviously modulate the perioperative immunity of patients, which was the possible mechanism underlying the anesthesia-induced recurrence and metastasis of tumors after surgery [7, 27, 28]. Dexmedetomidine (DEX) is a sedative used widely in tumorectomy anesthesia and postoperative analgesia. However, recently, Ueshima reported that DEX negatively modulated the immunity of murine DCs by suppressing the phagosome proteolysis and migration of DCs [22]. This suggested that the perioperative use of DEX could affect the perioperative immunity and further affect the recurrence and metastasis of tumors after surgery. DCs and CTLs are both important immune cells against tumor cells. In order to investigate the effects of DEX on the perioperative immunity of patients, we detected the effects of DEX on human DCs and CTLs. We found that (1) DEX inhibited maturation and function of human DCs (Fig 2), and (2) DEX-treated DCs could inhibit the immune function of CTLs (Fig 3). These showed that DEX could inhibit the perioperative immunity of patients, in line with the report of Ueshima [22]. Further study showed that human DCs expressed the α2-adrenoreceptors (Fig 4), the DEX targeted receptor. And α2-adrenoreceptor inhibitor yohimbine partly reversed the inhibitory effects of DEX on DCs and co-cultured CTLs (Figs 2 and 3). This proved that DEX modulated perioperative immunity mainly by α2-adrenoreceptors. Previous study indicated that ERK1/2, AKT and p38 were the main downstream signals of murine α2-adrenoreceptors [26, 29–31]. Thus we also detected the effects of DEX on the ERK1/2, p38 and AKT in human DCs. Interestingly, ERK1/2 and AKT were both activated in DEX-treated DCs, which was inhibited by α2-adrenoreceptors antagonist yohimbine (Fig 5). ERK1/2 inhibitor and AKT inhibitor also blocked the effects of DEX on the human DCs, respectively (Fig 5). These suggested that ERK1/2 and AKT were the main downstream signals molecules of DEX activated α2-adrenoreceptors in DCs.

Previous study showed that venous infusion with DEX reduced the plasma levels of proinflammatory cytokines such as TNF-α and IL-6 in rats [37]. Brain surgery patients may also benefit from DEX for its neuroprotective effect [38]. These above informations are contrary to the DEX’s immunosuppression of our data, suggesting the diverse functions of DEX during different pathological conditions. Regretfully, we detected only the effects of short-term treatment of DEX on human DCs. The long-term effects of DEX exposure on the DCs remain unclear. In addition, the dose choice of DEX in cultured cells is based on the clinical plasma concentration, but the real condition of DEX treatment on the immunity of tumor patients need further confirmation.

Briefly, our data showed that dexmedetomidine could inhibit immunity of cultured human dendritic cells via activating of α2-adrenoreceptors and its downstream signals ERK1/2 and AKT. The perioperative use of dexmedetomidine should be careful in tumor patients.

## Supporting Information

S1 Table Effect of DEX on IL-12 and IL-23 in mature DCs. (DOCX)

S2 Table Effects of DEX-treated DCs on co-cultured CTLs proliferation and IFN-γ protein. (DOCX)

S3 Table Effects of AKT, ERK1/2 inhibitors on production of IL-12 p35, IL-12 p40 and IL-23 p19. (DOCX)
