# Supplementary material for: Dexmedetomidine Inhibits Maturation and Function of Human Cord Blood-Derived Dendritic Cells by Interfering with Synthesis and Secretion of IL-12 and IL-23
Source: PLoS One. 2016 Apr 7;11(4):e0153288. doi: 10.1371/journal.pone.0153288 (PMC4824534; doi:10.1371/journal.pone.0153288)
Supplement: S1 Table — (DOCX) [file pone.0153288.s001.docx]

**Supplementary Table 1:** Effect of DEX on IL-12 and IL-23 in mature DCs. (mean+SD)

| Groups | mDCs | mDCs+1ng/ml DEX | mDCs+1ng/ml DEX+4ng/ml YOH | mDCs+2ng/ml DEX | mDCs+2ng/ml DEX+8ng/ml YOH | mDCs+4ng/ml DEX | mDCs+4ng/ml DEX+16ng/ml YOH |
| --- | --- | --- | --- | --- | --- | --- | --- |
| IL-12 p35 mRNA | 1.00+0.01 | 0.81+0.07* | 1.40+0.02^△^ | 0.19+0.01* | 0.39+0.02^▲^ | 0.11+0.01* | 0.14+0.01^●^ |
| IL-12 p40 mRNA | 1.00+0.11 | 0.33+0.03* | 3.79+0.12^△^ | 0.74+0.05* | 1.65+0.02^▲^ | 0.20+0.00* | 0.33+0.03^●^ |
| IL-23 p19 mRNA | 1.00+0.07 | 0.40+0.04* | 0.51+0.05^△^ | 0.32+0.02* | 2.11+0.03^▲^ | 0.14+0.01* | 0.26+0.01^●^ |
| IL-12 protein (pg/ml) | 87.66+3.18 | 81.57+11.44* | 98.80+7.35^△^ | 77.80+4.49* | 85.41+0.23^▲^ | 63.47+1.74* | 70.81+1.55^●^ |
| IL-23 protein (pg/ml) | 181.12+34.94 | 90.20+31.64* | 245.77+42.41^△^ | 87.54+22.22* | 159.11+84.98^▲^ | 150.85+25.74* | 209.75+34.62^●^ |

**Note:** mDC: control group of mature DCs; DEX: dexmedetomidine; YOH: yohimbine. * *P*<0.05, compared to mDCs group; ^△^ *P*<0.05, compared to mDCs plus 1ng/ml DEX group; ^▲^ *P*<0.05, compared to mDCs plus 2ng/ml DEX group; ^●^ *P*<0.05, compared to mDCs plus 4ng/ml DEX group.
