# Supplementary material for: Dexmedetomidine Inhibits Maturation and Function of Human Cord Blood-Derived Dendritic Cells by Interfering with Synthesis and Secretion of IL-12 and IL-23
Source: PLoS One. 2016 Apr 7;11(4):e0153288. doi: 10.1371/journal.pone.0153288 (PMC4824534; doi:10.1371/journal.pone.0153288)
Supplement: S2 Table — (DOCX) [file pone.0153288.s002.docx]

**Supplementary Table 2:** Effects of DEX-treated DCs on co-cultured CTLs proliferation and IFN-γ protein. (mean+SD)

| Groups | CTLs+mDCs | CTLs+mDCs+1ng/ml DEX | CTLs+mDCs+1ng/ml DEX+4ng/ml YOH | CTLs+mDCs+2ng/ml DEX | CTLs+mDCs+2ng/ml DEX+8ng/ml YOH | CTLs+mDCs+4ng/ml DEX | CTLs+mDCs+4ng/ml DEX+16ng/ml YOH |
| --- | --- | --- | --- | --- | --- | --- | --- |
| MTS assay  (OD value) | 0.686+0.053 | 0.556+0.048* | 0.664+0.040^△^ | 0.502+0.044* | 0.576+0.025^▲^ | 0.405+0.003* | 0.466+0.016^●^ |
| IFN-γ levels (pg/ml) | 42.18+4.08 | 31.04+5.09* | 45.80+3.71^△^ | 33.97+5.47* | 48.38+5.58^▲^ | 27.19+7.24* | 40.84+0.68^●^ |

**Note:** CTLs+mDCs: control group of co-cultured CTLs and mature DCs; DEX: dexmedetomidine; YOH: yohimbine. * *P*<0.05, compared to CTLs+mDCs group; ^△^ *P*<0.05, compared to CTLs+mDCs plus 1ng/ml DEX group; ^▲^ *P*<0.05, compared to CTLs+mDCs plus 2ng/ml DEX group; ^●^ *P*<0.05, compared to CTLs+mDCs plus 4ng/ml DEX group.
