# Supplementary material for: Dexmedetomidine Inhibits Maturation and Function of Human Cord Blood-Derived Dendritic Cells by Interfering with Synthesis and Secretion of IL-12 and IL-23
Source: PLoS One. 2016 Apr 7;11(4):e0153288. doi: 10.1371/journal.pone.0153288 (PMC4824534; doi:10.1371/journal.pone.0153288)
Supplement: S3 Table — (DOCX) [file pone.0153288.s003.docx]

**Supplementary Table 3:** Effects of AKT, ERK1/2 inhibitors on production of IL-12 p35, IL-12 p40 and IL-23 p19. (mean+SD)

| Groups | mDCs | mDCs+2ng/ml DEX | mDCs+2ng/ml DEX+LY294002(30μM) | mDCs+2ng/ml DEX+PD98059(40μM) |
| --- | --- | --- | --- | --- |
| IL-12 p35 | 1±0.34 | 0.08±0.02* | 0.66±0.27^△^ | 2.06±0.38^△^ |
| IL-12 p40 | 1±0.03 | 0.54±0.10* | 2.71±0.50^△^ | 0.60±0.10 |
| IL-23 p19 | 1±0.14 | 0.79±0.12* | 0.47±0.12 | 2.11±0.21^△^ |

**Note:** mDCs: control group of mature DCs; ***** *P*<0.05, compared to mDCs group; ^△^ *P*<0.05, compared to mDCs plus 2ng/ml DEX group.
